# What is stopping us? An implementation science study of kangaroo care in British Columbia’s neonatal intensive care units

**DOI:** 10.1186/s12884-020-03488-5

**Published:** 2021-01-12

**Authors:** Sarah Coutts, Alix Woldring, Ann Pederson, Julie De Salaberry, Horacio Osiovich, Lori A. Brotto

**Affiliations:** 1 Perinatal Services BC, 260 – 1770 W 7th Ave, Vancouver, BC V6J 4Y6 Canada; 2 BC Women’s Hospital, 4500 Oak Street, Vancouver, BC V6H 3N1 Canada; 3 University of British Columbia, 2329 West Mall, Vancouver, BC V6T 1Z4 Canada; 4 Women’s Health Research Institute, H214 – 4500 Oak Street, Box 42B, Vancouver, BC V6H 3N1 Canada; 5 Department of Obstetrics and Gynaecology, University of British Columbia, 2775 Laurel Street, Vancouver, BC V5Z 1M9 Canada

**Keywords:** Kangaroo care, Kangaroo mother care, Neonatal intensive care unit, Implementation science, Neurodevelopment, Family-centered care, Canada

## Abstract

**Background:**

The goal of the Neonatal Intensive Care Unit (NICU) is to provide optimal care for preterm and sick infants while supporting their growth and development. The NICU environment can be stressful for preterm infants and often cannot adequately support their neurodevelopmental needs. Kangaroo Care (KC) is an evidence-based developmental care strategy that has been shown to be associated with improved short and long term neurodevelopmental outcomes for preterm infants. Despite evidence for best practice, uptake of the practice of KC in resource supported settings remains low. The aim of this study was to identify and describe healthcare providers’ perspectives on the barriers and enablers of implementing KC.

**Methods:**

This qualitative study was set in 11 NICUs in British Columbia, Canada, ranging in size from 6 to 70 beds, with mixed levels of care from the less acute up to the most complex acute neonatal care. A total of 35 semi-structured healthcare provider interviews were conducted to understand their experiences providing KC in the NICU. Data were coded and emerging themes were identified. The Consolidated Framework for Implementation Research (CFIR) guided our research methods.

**Results:**

Four overarching themes were identified as barriers and enablers to KC by healthcare providers in their particular setting: 1) the NICU physical environment; 2) healthcare provider beliefs about KC; 3) clinical practice variation; and 4) parent presence. Depending on the specific features of a given site these factors functioned as an enabler or barrier to practicing KC.

**Conclusions:**

A ‘one size fits all’ approach cannot be identified to guide Kangaroo Care implementation as it is a complex intervention and each NICU presents unique barriers and enablers to its uptake. Support for improving parental presence, shifting healthcare provider beliefs, identifying creative solutions to NICU design and space constraints, and the development of a provincial guideline for KC in NICUs may together provide the impetus to change practice and reduce barriers to KC for healthcare providers, families, and administrators at local and system levels.

## Background

The goal of the Neonatal Intensive Care Unit (NICU) is to provide optimal care for preterm and sick infants while supporting their growth and development. Preterm infants are neurobehaviourally immature with their early birth representing significant trauma during a sensitive period of growth and maturation. The extrauterine environment plays a major role in shaping the neurodevelopmental trajectory of the infant when development continues outside the protective environment of the womb [1]. The NICU is a stressful sensory environment for preterm infants and often cannot adequately support the preterm infant’s neurodevelopmental needs. Kangaroo Care (KC) is an evidence-based developmental care strategy that has been shown to be associated with improved short and long term neurodevelopmental outcomes and is known to decrease the stress of the NICU environment [2, 3].

Despite significant advances in neonatal intensive care which have improved the survival of preterm infants, these infants continue to be at a high risk for neurodevelopmental delays [4, 5]. The adverse sensory environment of the NICU (i.e. loud noises, bright lights, noxious odors, procedural touch, and pain) are vastly different from the intrauterine environment that regulates sensory input and stimulation by the movement of the mother’s body and fetal activity within warm amniotic fluid. This mismatch influences physiological processes and pathways and may predispose the infant to later disease and poorer developmental outcomes [6].

Admission to the NICU often involves early separation of the infant from their parents. Separation from the mother at birth disrupts the evolutionary biological expectancies of social engagement which are critical for co-regulation and healthy development of both the infant and mother [7]. Preterm birth disrupts immediate postpartum care practices that support mother-infant togetherness and co-regulation such as placing a newborn on the mother’s chest. Instead the infant is exposed to noxious stimuli, immediate handling, and sometimes painful procedures. An incubator is the standard location of care and substitute for the maternal care environment; this and other forms of separation disrupt the establishment of the early maternal infant relationship. After birth, close physical contact with the newborn is crucial for the parent and infant to attach and develop a secure attachment relationship [8]. Negative neurologic outcomes can be reduced through positive maternal–infant interactions during the NICU hospitalization and once at home. Emotional and physical closeness has the potential to maximize positive neurodevelopmental outcomes in preterm infants [9].

For parents, the birth of a preterm or sick infant and subsequent admission into the NICU can be a traumatizing experience filled with feelings of anxiety, guilt, lack of confidence in their parenting skills, and difficulty bonding and attaching to their infant as they learn to become parents in an unfamiliar and intimidating environment [10, 11]. Family-centered care (FCC), a philosophy of care in which families, parents, and healthcare providers (HCPs) embrace a partnership, has been shown to increase parental presence and participation in care [12]. A philosophy of FCC recognizes parents’ unique role in the care of their baby and positions parents as part of the infant’s health care team. FCC is team-oriented and multidisciplinary and involves the families in breastfeeding, KC, infant care planning, and unrestricted presence with their infant [12]. FCC represents a paradigm shift away from the traditional health care expert model to a model that recognizes the essential role of parents in contributing to positive health outcomes for the infant and the family. There is general consensus that the movement away from a purely medical approach towards one that supports parent involvement in the care of their hospitalized infant is vital. However, the degree to which FCC is adopted and implemented in NICUs varies across institutions, countries, and regions [13].

One element that is widely accepted as a practice within FCC is KC. Parent-delivered interventions, such as KC, support the expression of FCC philosophies within an NICU. The Kangaroo Mother Care (KMC) model, first introduced in the 1970s by pediatrician Edgar Rey in Bogotá, Colombia, presented a drastic shift in neonatal care in which the mother’s chest became the place of care in order to address the numbers of infants who were dying due to infection and hypothermia related to staff and equipment shortages [14]. A similar shift in neonatal care—the introduction of Humane Care from Tallinn, Estonia in which mothers became the primary caregivers of their babies—was introduced due to nursing shortages [15]. In KMC and the Humane Care models, mothers are trained to provide nearly 24-h care and the mother’s chest serves as the place of care. Models of care which explicitly incorporate parents in the care of their infant in the NICU are now well supported in the literature [16]. Progression and integration of FCC and KMC has been slow, however; both need appropriate facilities and resources, policies, education for parents and HCPs, and shifts in beliefs and attitudes [17].

KMC provides early and continuous skin-to-skin contact in the kangaroo position between an infant and a parent, ideally for 24 h a day, with exclusive breastfeeding and proper follow-up at home and in the community [18, 19]. Terms such as skin-to-skin care/contact, KMC, and KC are used interchangeably to describe this model. In our setting we chose to use the term Kangaroo Care to signal that others besides a mother can provide an infant this care. KC offers an environment that reduces stress and provides comfort, support, and positive social interaction for both parents and infants [20, 21]. KC has been shown to facilitate an infant’s transition to extrauterine life, to promote early parent-infant attachment and bonding, to enhance breastfeeding, to decrease pain responses and risk of infection, to have positive effects on infant sleep patterns, to promote neurodevelopment, and regulate physiology and behaviour in the short- and long-term [19, 21]. KC is recommended as a feasible, natural, cost-effective, and evidence-based intervention that should be a standard of care for all infants, regardless of geographic location or economic status [22].

In resource-supported settings like Canada, KC is seen as a complement to conventional NICU care in which infants spend most of their time in an incubator and continuous KC is not common. Conventional medical care protocols often result in incubator-based treatments that require maternal separation from preterm infants [23]. KC, if practiced, is most often implemented intermittently, for a limited time period when the infant is considered stable, on average for one to 2 h at a time [24]. Despite the strong evidence for positive infant, maternal, and family outcomes and recommendations for the widespread adoption of KC internationally, the integration of continuous KC into the care of preterm infants remains extremely variable [25, 26].

Approximately 11% of babies [27] in British Columbia (BC) are born preterm and require care through BC’s publicly funded healthcare system, which serves a demographically diverse population of approximately 5.1 million people spread over 944,735 km^2^. The BC Ministry of Health and Ministry of Child and Family Development identified supporting studying KC implementation in the province as a priority due its documented positive effects on short and long-term child health outcomes and reduction of maternal stress, anxiety, and depression by supporting parent-infant bonding and providing an environment that enhances cognitive and emotional development [19]. This study aimed to explore the barriers and enablers to the practice of KC to enhance the uptake of KC in the province’s NICUs.

Despite the known health benefits, global recommendations and substantial body of evidence, there are documented barriers to universal and widespread adoption of KC. We expected that KC practices in BC would vary by NICU and that it would be unknown how often KC occurs as most NICUs do not document or track KC on a regular basis. Previous research on the barriers and enablers to the implementation of KC has focused both on the health system and HCP perspectives and the majority has been done in low- and middle- income countries. Given the different context of care, recommendations from international studies are not easily translatable to the Canadian context. In high income settings barriers to KC include the lack of staff, education and training, the absence of policies and guidelines, staff uncertainty about the importance of KC and the perception that KC is a strain for mothers [29–31]. It has been suggested that effective KC implementation relies on the health system and HCPs to overcome these barriers and harness the enablers that exist within each facility or unit [32].

There is a need to better understand the barriers that prevent full scale adoption of KC in order to implement it more broadly. To do so, we focused on the experiences of HCPs across 11 NICUs in BC. The Consolidated Framework for Implementation Research (CFIR) guided our research methods. The CFIR is a practical conceptual framework often used to assess potential barriers and enablers to the implementation of an innovation. The CFIR guided our project to identify and understand the contextual factors that exist in NICU settings [33]. The CFIR is comprised of 5 broad domains: (1) intervention characteristics, (2) outer setting, (3) inner setting, (4) individual characteristics, and (5) process. These domains guided our research methods in terms of structuring the data collection methods, analysis, and reporting of findings [34]. The results of our study will inform future research on facility-based implementation strategies and recommendations for implementing KC consistently in BC.

## Methods

### Design

Our methods, grounded in implementation science, aimed to identify the gaps in KC practice at the HCP, unit, and healthcare system level [35]. Implementation research aims to identify the factors that function as barriers and enablers to specific interventions [34]. Our study design was also informed by the extensive research carried out by Anne-Marie Bergh and colleagues to implement and scale-up KC throughout healthcare institutions in Africa and Asia [36–38].

### Setting

**Fig. 1:**
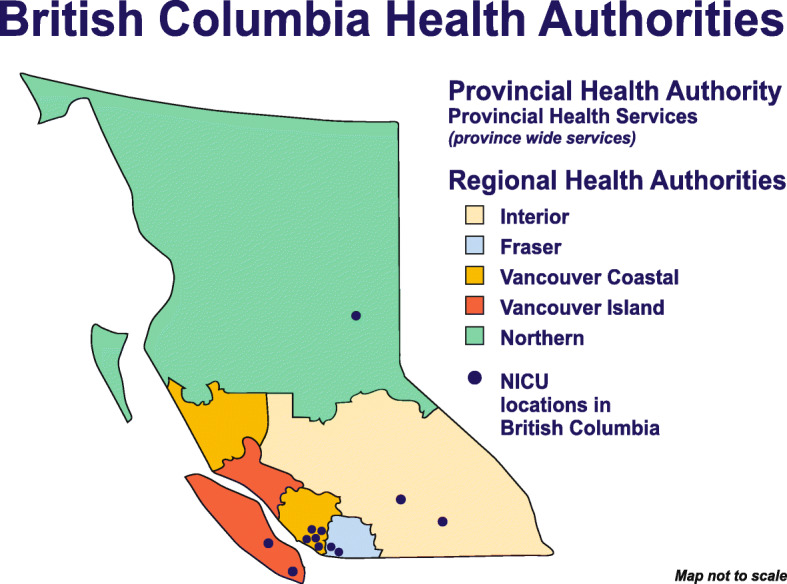
Map of British Columbia and geographical location of NICUs

This study was conducted in 11 of the 13 NICUs in BC (two declined to participate). The NICUs ranged in size from 6 to 70 beds; with mixed levels of care from less acute neonatal care to the most complex neonatal acute care. Table 1 outlines the level of care, number of funded beds, and geographic location of the 11 NICUs in this study. Figure 1 shows the geographic location of BC NICUs indicating that they are predominantly clustered in the lower mainland of the province, where population density is highest. However, several NICUs are located in lower density areas and serve as regional hubs.

**Table 1:** Description of NICUs

Hospital identifier	Level of care	Number of funded beds	Location
A	2b	10	Northern
B	2b	12	Interior
C	2b	8	Interior
D	2a	6	Vancouver Island
E	3+	22	Vancouver Island
F	3+	70	Provincial Health
G	3+	32	Fraser
H	3+	24	Fraser
I	2a	6	Vancouver Coastal
J	2a	6	Vancouver Coastal
K	2a	6	Vancouver Coastal

Footnote: Refer to the additional file attached for the ‘Neonatal Daily Classification Tool’ which outlines the NICU Levels of Care

All NICUs in BC have a variety of HCP roles depending on the size and level of patient acuity. NICU nurses in BC are required to complete neonatal specialty training prior to being hired; this training includes an introduction to KC. Three of the NICUs were Single Family Room (SFR) design that included a bed or couch for parents to stay overnight. One NICU was a hybrid design with a combination of SFR, semi-private rooms, and open-bay spaces, and the remaining seven NICUs were exclusively open-bay design with six to eight infant beds per room and usually no place for parents to stay overnight. KC practices in each NICU were unknown to the research team prior to the site visit. At the beginning of the study, few NICUs collected and reported data on KC. As there is no provincial data registry that records information on KC practice by NICU, we also gathered information on the extent of current KC practices at each site during the interviews.

### Participants and sampling

Perinatal leaders of each regional health authority were invited by Perinatal Services BC to join the study. NICU managers and educators at each participating hospital were identified as potential interview subjects. Participation was voluntary. The researcher (SC) contacted each NICU to arrange a convenient time for the research team to visit. Recruitment posters outlining the study and information about the site visit were displayed on NICU notice boards in each unit to inform staff. In most units NICU educators or a delegate coordinated site study activities and liaised with the research team. A purposive sampling approach was employed to recruit participants to achieve a multidisciplinary sample of HCPs who were knowledgeable about the day to day KC practices in each unit [39, 40]. Prospective interview participants were recruited by the NICU educator or unit manager. The availability of HCPs balanced with the size of the NICU determined sample size.

### Data collection

Face-to-face, semi-structured interviews were conducted to allow topics to be explored in sufficient depth yet provide the flexibility to elicit ideas not anticipated at the outset of the research [28]. Semi-structured interviews encouraged the sharing of the participants’ assumptions, feelings, perceptions, and opinions about KC and offered the space for participants to construct their own “framework of meanings” [28].

The interview guide was informed by the research question, review of the literature, and established quantitative measures, and questions and surveys previously used by comparable studies in other countries [29, 41–43]. The research team reviewed the interview guide for content and flow, and trialed the guide for length of time and appropriateness of the questions. Two interviewers conducted all the HCP interviews. One interviewer (SC) is a nurse with a background in neonatal nursing, a graduate degree, and experience in qualitative interviews. The second interviewer (AW) has experience in qualitative research and interviewing, and a social science graduate degree.

Before the start of the interview, HCPs were informed that any personal information provided would be kept confidential, anonymized, and that their participation in the study was entirely voluntary. All participants provided written informed consent. Interviews took place in the hospital, and were audio recorded and transcribed by a professional transcriptionist who removed all direct and indirect identifiers. Interviews lasted between 30 to 45 min and took place during the HCP’s work time. No compensation was offered for participation. In some hospitals, time constraints, combined with a lack of private space, prevented interviews from being recorded; in these cases detailed interview notes were taken and used to confirm codes and themes during subsequent analysis of the interviews.

Interviews were conducted between August 2018 and March 2019. Face-to-face interviews focused on HCPs’ current practices, the NICU environment, resources, and their experiences and attitudes regarding KC and supporting families on the unit. Table 2 provides examples of interview questions used. The complete interview guide is attached as a supplementary appendix.

**Table 2:** Example healthcare provider interview prompts

Topic	Prompts
Knowledge about Kangaroo Care	How familiar are you with Kangaroo Care? How did you come to learn about it? How is Kangaroo Care introduced to parents in the NICU?
Barriers to Kangaroo Care	What barriers do you see at this hospital in terms of parents engaging in KC? How feasible is 24/7 KC in this setting? If it isn’t feasible, what would need to change to support it?
Enablers to Kangaroo Care	How do you see parents being supported or enabled in their efforts to provide KC? What do you think is working well to facilitate KC at this hospital? What kind of awareness and educational activities do you have in your facility to introduce KC to staff members?

The study was approved by the Behavioural Research Ethics Board at the University of British Columbia and the Harmonized Research Ethics Boards of the participating hospitals in each health authority.

### Data management and analysis

Two members of the research team (SC, AW) conducted conventional qualitative content analysis of the data to maximize understanding of the current state of KC practice by capturing context using participants’ unique perspectives [44]. Initially, each analyst read a random selection of the transcripts independently and identified factors that influenced the practice of KC in each NICU. An inductive approach and open coding [45] facilitated identifying themes in the data. Coding discrepancies were discussed and resolved with a third member of the research team (AP). Once no new themes emerged, the coding scheme was finalized by the research team (SC, AW, AP, HO, JDS, LB) and applied to the remaining transcripts. Field notes were kept to record researcher observations of the physical features of each NICU (e.g., number of beds, space) and to document available resources (e.g., equipment, amenities for families). Transcripts were coded using NVivo Version 10 qualitative analysis software.

## Results

We conducted a total of 35 recorded HCP interviews from 11 NICUs. Participants included: Neonatologists (3), NICU nurses (9), Nurse Educators (9), NICU clinical managers (5), Occupational Therapists (3), and a Pediatrician (1), Midwife (1), Respiratory Therapist (1), Dietician (1), Physical Therapist (1) and Lactation Consultant (1).

Four overarching themes were identified from HCPs’ descriptions of the barriers and enablers to KC encountered in each setting: 1) the NICU physical environment; 2) healthcare provider beliefs about KC; 3) clinical practice variation; and 4) parent presence. Each theme contains several sub-themes. Depending on the specific features of a given site, themes could function as either an enabler or a barrier to practicing KC.

### The NICU physical environment

- Physical space of the NICU

The majority of BC NICUs reported inadequate space, such as limited space for a comfortable reclining chair alongside the incubator and other equipment while maintaining a sense of privacy for the family as a barrier to practicing KC. Each NICU had a unique footprint and infant care spaces varied but facilities had similar requirements for specialized equipment. There were differences in how units coped with their footprint. For example, some NICUs could comfortably fit a reclining chair beside each infant incubator or crib whereas others needed to move chairs in and out of the space or made room by moving other equipment or the infant bed out of the way. An Occupational Therapist described the challenges of providing sufficient space for KC and maintaining some level of privacy.

“…When the NICU is full, I think that sometimes the juggling of trying to get around each of the... little spaces with lots of parents that are there, [parents] still want to have a little bit of privacy as well.”

b. Kangaroo Care chairs

The most tangible barrier to prolonged KC reported was the lack of comfortable, reclining chairs. There was much variation in the type and number of chairs in each NICU. Most NICUs did not have a chair for every family and most chairs did not recline. It was reported that the purchase of KC chairs was typically not included in the operational budget. One NICU recently initiated a campaign for the purchase of KC chairs through its hospital foundation and new chairs were purchased through charitable donations. Some HCPs expressed distress over the lack of appropriate chairs to adequately support KC:

“… Some of our other chairs had broken, and we didn't actually have enough chairs for all the parents to sit. So we had to run up to the maternity ward and bring stuff down. It was embarrassing.” (Nurse Educator)

iii. Single family room NICUs

HCPs suggested the presence of a bed or couch signals the importance of parental presence in the NICU to the family and facilitates parents staying overnight. In general, HCPs believed that parents value the privacy and comfort provided by the ability to adapt the room to feel like home. HCPs from two NICUs reported that when they transitioned from an open-bay facility to a SFR NICU parental presence increased. However, HCPs from one SFR NICU reported that though there was a gradual increase in parental presence following the transition to SFRs, overall the parental presence remained low. Some units with SFR commented that even when amenities for families were not perfect, families could still be present. In theory, HCPs believed that SFRs should better support KC practices. SFR NICUs are better designed to support parent presence, offer privacy and had fewer barriers to supporting KC. For example, one Physiotherapist said,

“...We have a really good situation where we’ve got individual rooms. Ideally we need a bathroom in each room but we don’t have that. [despite that implied] some of the families will be here 24/7.”

Participants, predominantly those working in the lower acuity sites, voiced concerns over scarcity of resources. HCPs in these sites focused on the environmental barriers in the open bay NICUs and described Level 3 NICUs as having “more resources than us,” particularly with regard to KC chairs and SFRs. Despite disparity in resources, HCPs in all NICUs generally believed they were maximizing KC within the limits of what was possible in their unit.

### Healthcare providers’ beliefs about kangaroo care

iv. Belief in the value of Kangaroo Care

HCPs’ attitudes towards KC were positive and supportive of KC as an intermittent practice but concerns surfaced when HCPs were asked how KC could be supported for longer than 1 to 2 h a day. Concerns included HCPs’ perceptions of parents’ ability or willingness to stay longer, whether longer KC truly produced better outcomes, discomfort regarding having parents present throughout the day or night, and whether infants could tolerate longer time in KC. HCPs across NICUs noted that the value and belief in the efficacy of KC by nurses is critical in determining whether nurses will adjust their practice to maximize KC. We observed varied beliefs in the perceived value of KC between HCPs, within the same NICU, and between NICUs in BC:

“...So much of this relies on the nurses and how they sell it, and the importance they place on it ...because there's so many different people and personalities and I think still in many ways, having babies in a crib or a bassinet is easier for nurses than having them attached to a mom” (Occupational Therapist).

Several NICUs identified KC ‘champions’ within their units who were individuals known to be exceptionally supportive of the practice. Champions were viewed as important mobilizers to advance KC as the standard of care in their respective NICUs. Champions often worked alone and in isolation and were advancing KC practice without the support of policy, guidelines, and/or other members of the healthcare team. A few NICUs identified that the introduction of new staff members from higher acuity NICUs or from other countries where KC was a standard practice helped to influence the implementation of KC. The new staff members were able to demonstrate ways that KC could work in any environment and helped to normalize the practice.

e. Belief in the local feasibility of Kangaroo Care

Some nurses were doubtful of the practicality of providing prolonged and continuous KC due to environmental restrictions (e.g., NICU footprint and design) and resource limitations (e.g., availability of chairs, staffing). The inability to overcome these limitations led to a common attitude that ‘it may be best practice, but it is not possible in this NICU’. HCPs in Level 2 NICUs or open-bay designs were more likely to believe that, if the physical environment of the NICU proved impractical for the delivery of prolonged KC, the degree of HCP support for it was irrelevant.

f. Understanding of the Kangaroo Care model

HCPs reported hearing about KC at different stages of their formal education and career. Some sites had more opportunities for paid staff education, but other sites were challenged to disseminate education when staff were not paid to attend education sessions or were required to attend on a day off. While all HCPs reported having a baseline understanding of KC, knowledge of the efficacy and scientific evidence supporting KC varied considerably. NICU educators reported providing additional education sessions to staff to improve KC practices to address such knowledge gaps.

Nurses most frequently cited improved immediate infant physiological outcomes such as stabilized heart and breathing rates, increased maternal milk supply, and parent-infant bonding but rarely described long-term positive health outcomes for infants and mothers:

“We’ve done an okay job with nursing to really help them understand that skin-to-skin isn’t just a warm and fuzzy, it’s actually therapeutic both for the baby and for the family” (Nurse Educator).

The conflation of “Kangaroo Care” with “skin-to-skin care” was a barrier to KC and was nearly universal among participants; the two terms were often used interchangeably. In general, HCPs were more comfortable using the term skin-to-skin care. Only a few HCPs used the term Kangaroo Care to describe the holding of an infant skin-to-skin in the NICU. Most often these HCPs noted that they had personally read about KC in a journal article or attended an education session about KC. As a NICU manager said, “We do Kangaroo Care, but I don’t know that it’s officially Kangaroo Care. We encourage skin-to-skin.”

Similarly, the lack of documentation in half of the NICUs was noted as a barrier to the understanding of KC as a model of care. When asked about how KC could be strengthened in their unit, a Neonatologist noted,

“The other distinction we don't make well enough here and you can see it in our flow sheets is it's not even really clear the difference between skin-to-skin versus holding and cuddling…We can't record if somebody's embracing a philosophy if we don't [record it]-- because that is a philosophical difference, right?”

g. NICU routines

Every NICU has its own set of routines or practices that help HCPs organize their day and influence how and when care is provided. When asked about barriers and enablers in the NICU to KC, HCPs often talked about daily routines that made it difficult to support prolonged KC such as daily lab work, routine handling times for infants, shift changes, handovers, breaks, and patient rounds. Set parent “visiting” hours and patient care rounds were the two routines frequently noted as difficult to change. Some HCPs described a belief that KC was one of the most important things parents can do with their infant and that this was part of their ‘daily routine’ always offered to families. Other HCPs reported KC as a practice that happened if they had enough time and that it was often last on the daily list of nursing care activities:

“And sometimes when there’s more cords [attached to the baby for monitoring] or [you are] trying to get mom on your schedule, it’s like I have two other babies to see so I have to feed him this time. I’m sorry, I can get you tucked in now [set up in KC] otherwise I’m in the middle of something else” (Nurse).

### Clinical practice variation

h. Individual care practices and HCP comfort levels

Within each NICU we observed that HCPs’ approach toward KC was not consistent for all infants. Clinical practice variation refers to infants receiving differing care depending on when, where, and by whom they are being cared for, despite evidence for best practice. One HCP noted that “… some HCPs practice Kangaroo Care consistently and others not so much… the narrative in the story about it is told differently depending on who you are talking to” (Pediatrician).

Some nurses voiced concerns about the safety and appropriateness of KC for all infants. HCPs reported KC was denied or delayed based on an individual nurse’s levels of comfort with handling infants that are small and considered more vulnerable or noted as unstable and their confidence and technical skills to transfer an infant to a parents’ chest when the infant is attached to lines and equipment: “Yeah, because if God forbid you take that kid out to do something that’s theoretically great and then you break the baby” (Physiotherapist).

Nurses reported being fearful of dislodging tubes, extubating, pulling out central lines and intravenous catheters, or causing instability in the infant when transferring an infant to KC. KC was thought of as a more risky intervention than having the infant in the incubator: “So if all of a sudden I had a sick baby -- I’d be nervous to take it out. Nobody wants to be the one to dislodge those kinds of tubes [respiratory support tubes]” (Nurse).

HCPs also noted varying definitions and criteria for which infants in their unit were eligible for KC. There was no consensus among all the NICUs on the eligibility criteria for KC; each NICU had its own set of rules or policy guidelines. Unit practice norms, combined with the HCPs’ personal comfort, confidence, and skills, informed their beliefs about which infants were able to be held in KC. Nurses from Level 2 NICUs were more likely to be uncomfortable with infants on higher respiratory support (such as continuous positive pressure) and with central line catheters (umbilical venous and arterial catheters). Several nurses from Level 2 NICUs reported completing practicums in Level 3 NICUs where some KC practices were more established such as KC with CPAP. These nurses reported that observing KC with infants on CPAP was helpful in building comfort and confidence. They found when they returned to their home NICU, however, this practice did not occur as not enough staff were comfortable and it was not considered a standard of care:

“…This is probably a big barrier being a level 2, we don’t get a lot of vented babies or UV lines. So comfort level with positioning a baby and taking a baby out of an incubator with all of those lines can sometimes probably be a barrier. I don’t take the vented babies out yet” (Nurse).

HCPs in all NICUs reported lack of time and heavy workloads as barriers to supporting KC. Setting up infants and parents to do KC was viewed as a time and resource-demanding task. Time constraints, competing priorities, staffing levels, and availability of technical expertise and support in the NICU, such as the availability of respiratory therapists, affected the practice of KC. The perceived investment of time and the impact on workload increased with the acuity of the infant in all NICUs.

Nurses often behaved as ‘gatekeepers’ to the scheduling of infant care, parent involvement, and KC. HCPs in many sites described ‘leaving babies to rest’ or ‘undisturbed’ between routine feeding times. The practice of ‘clustering care’, in which nurses group an infant’s care such as diaper changes, position changes, and temperature-taking together instead of spacing them out over time in order to maximize rest or sleep time, was noted as conflicting with prolonged and continuous KC. HCPs reported organizing their shift according to handling times for the infant. When a parent came in for KC during a planned ‘undisturbed’ time or what nurses felt was rest time, parents were prevented from holding their infant in KC until the designated handling time. Most often this correlated with the infant’s feeding times, for example every 3 h. When asked whether infant care could be integrated into KC time, most nurses noted they had not considered doing this or noted that disturbing the infant’s rest for a parent to hold in KC seemed counterintuitive. HCPs worried about causing instability and disrupting sleep patterns if an infant was disturbed and placed on the parent’s chest. In sum,

“If you can work on a model where the parents are more in charge of the care of their baby, then you reduce the workload on the staff. But as long as the staff are the gatekeeper to all of that, it’s going to feel like a lot of work for them” (NICU Manager).

i. Guidelines and documentation

Though several HCPs noted the importance of policy in supporting the uptake of KC and ensuring it was a standard of practice, only a few (3/11) NICUs had KC clinical policies or guidelines. One Nurse Educator noted that a recently implemented practice guideline helped to dispel myths and was a factor in practice change:

“When we did our clinical practice guideline, we tried to dispel the myth that, a baby who was intubated couldn’t go skin-to-skin… I think that putting that out there helped to dispel some of those historical practices that we were seeing, that were probably limiting skin-to-skin for babies and for families.”

However, as one HCP noted, the existence of a policy in a NICU did not in guarantee universal practice of KC: “We’ve done the policy and education so at some point we have to say this is important enough that people are held accountable if they’re not following hospital policy” (Lactation Consultant).

j. Multidisciplinary support

All HCPs stressed that NICU nurses were critical in leading and strengthening the practice of KC. The introduction of KC, transfer of information for families, and understanding of current practices were identified as a nurse’s responsibility: “I think that the – frontline bedside nurses are the biggest promoters and sometimes the biggest challenge” (Pediatrician).

Some HCPs reported the need for stronger multidisciplinary support to push the practice of KC. Nurses acknowledged their role in supporting KC but felt that there was a need for the entire NICU healthcare team to be on board in order to prioritize the practice. Patient rounds were suggested as a venue for multidisciplinary discussion about the logistics for a KC transfer, reinforcing it as a priority to families, and addressing the accountability of HCPs to assist families with KC:

“I think it has to be a team effort. So when you’re on rounds that should be a question that’s asked every single day. Have we [done Kangaroo Care] – where are we at? Are the parents aware of Kangaroo Care?” (NICU Manager).

### Parental presence in the NICU

k. Communication and messaging to parents

HCPs noted that the communication parents received about KC was critical in shaping their presence in the NICU. HCPs mentioned the importance of providing parents with correct communication on the evidence of the short-term stabilizing impact of KC and the long-term developmental impacts of KC; providing consistent information across HCPs and using multiple modes of communication (verbal, handouts, posters); and presenting KC as a treatment strategy.

Despite HCPs acknowledging the need for increased information-sharing, many HCPs said they were reluctant to communicate an expectation that parents be present if the NICU space and resources to support continuous KC were limited or unavailable. HCPs described feeling the need to ‘protect’ the parent and to balance encouraging KC with a perceived potential to elicit guilt and shame if parents were not able to be present in the NICU. HCPs consistently reported that parents had commitments outside the hospital and personal barriers to spending more time in KC. HCPs cited caring for siblings, work responsibilities, travel time, and postpartum pain and healing as parental barriers to KC. But,

“When I walk by a room and a baby’s lying in a cot alone I find that stressful. I immediately think “where’s the mother?” Not in a judgmental way. I don’t want to sound like I’m judging mothers. But more, ‘what messaging has this mother received that she’s not here?’” (Lactation Consultant).

xii. Adoption of family-centered care values

Integration of parents into the NICU through ensuring parents and infants were not separated, supporting parents to provide basic infant care, and assisting parents to become the expert on their infant was inconsistent across the NICUs. FCC principles were identified as important by a few HCPs but most HCPs did not use the term FCC. However, some HCPs recognized a recent shift in neonatal care towards an environment that is more welcoming toward parents and no longer treats parents as visitors but as partners in care. Yet one HCP felt concerned that they would be perceived as ‘lazy’ by parents if parents did the majority of infant care. Notably, none of the NICUs in BC had an explicit FCC policy.

HCPs were asked whether parents and infants were separated after birth, both on the maternity wards and when infants were transferred to the NICU. HCPs reported that separation still occurred after cesarean sections and when preterm and sick infants were taken to the NICU: “There is a lot of separation. Unnecessary separation in my opinion” (Nurse).

A third of NICUs (4/11) reported that their facility maintained visiting hours for families. Nurses working in NICUs that maintained such visiting hours justified the practice as a strategy to manage privacy and confidentiality during shift changes, handovers, and patient care rounds:

“The second thing is right now our nursery is closed 2 hours [a day] because [of] shift change and our colleagues are still concerned about confidentiality issues [during rounds and handover]” (Nurse).

Nurses working in NICUs with parent visiting hours held varied opinions on their appropriateness. Some nurses felt that it was difficult to change culture and other HCPs’ opinions and beliefs about parent presence during NICU routines like handover and rounds. Parent visiting hours were more likely to exist in NICUs with an open bay design.

xiii. Perceived supports for parental presence

Support for families to be present in the NICU varied highly. HCPs suggested several amenities that they perceived as supportive for parents’ presence in the NICU including SFR NICUs; paid accommodation close to the NICU; meals; paid parking; transportation vouchers; a family room for siblings (to address child care barriers); and a social worker to facilitate aspects of care in and out of the hospital. Given population density and distribution in BC, families and their infants are often transported to a NICU closer to home as the infant’s acuity decreases. HCPs noted that parents’ expressed frustration and surprise when they found the KC practices and resources or environment different between hospitals. If a family had a KC routine established in the first hospital, HCPs observed it was difficult to adapt to a new environment or the routine and culture of the new NICU. Language barriers and cultural practices of families were also cited as barriers to supporting KC. Many families were noted to have cultural restrictions, for example, a ‘30-day confinement period’, in which the mother traditionally stays at home. HCPs reported that it was difficult to convey the importance of KC to families who did not speak the same language as the HCP. Current KC resources for parents are only available in one language, English.

Several nurses noted that the ongoing opioid crisis in BC influenced parent presence in the NICU [46]. There has been a noted increase in newborns exposed to drugs in utero, some who have experienced Neonatal Abstinence Syndrome (NAS) symptoms and subsequent separation from their mothers when admitted to the NICU for observation. Many NICUs were unable to support rooming-in for these dyads. Nurses noted the stigma toward mothers who use substances, the lack of privacy in open-bay design NICUs, and the increased risk of the removal of infants from the birthing parent’s guardianship as barriers for parent presence in this population. Nurses also suggested that infants with NAS often needed to be held by HCPs or volunteers for long periods of time. It was suggested that promoting KC with this population would be beneficial to both the infants and mothers. Several sites requested the development of a practice guideline for KC for infants with NAS and for mothers who use substances.

HCPs across all NICUs identified that funded accommodation in close proximity to the NICU supported parents’ presence. Accommodation for families with infants in BC NICUs falls into four categories: 1) single rooms (four NICUs), 2) a room with a bed on the unit for families to sleep and rest for one to two nights when preparing for discharge (three NICUs), 3) a room available on the hospital campus provided through a hospital foundation or charity (three NICUs) and 4) no available accommodation (four NICUs). The distribution and availability of accessible accommodation for families across the province appears highly uneven.

“It is huge, and especially when we are one of the level three [NICUs] -- it makes me so sad that if you come down [travel] from wherever, and the only place to come is here, you are stuck here for six months, and you are paying out of your pocket [for accommodation]” (Nurse Educator).

## Discussion

This is one of the first studies to describe the practice of KC in Canada and specifically in BC. Interviews with HCPs from varied professional disciplines described the multifaceted factors that influence KC in BC. Our findings suggest that factors such as unit routine, clinical practices, and HCPs’ beliefs shaped the provision of KC across the NICUs to varying degrees, depending on other factors such as physical layout, the availability of reclining chairs, and parental presence.

An ongoing neonatal care paradigm shift is underway in all of the NICUs in this study. This paradigm shift has been described in the literature worldwide, as NICUs move from care focused on the healing of an infant’s medical problems toward a focus on parent participation in infant care and decision making. NICUs that are further along the path of this paradigm shift view the family as essential to achieving best outcomes for infants in the NICU [47].

KC in BC is caught in the midst of this global neonatal care paradigm shift and its evolution locally is not yet complete. While there was general awareness of the importance of KC as a parent-delivered developmental care intervention, the extent to which KC was happening and HCPs’ beliefs about KC varied widely, as did the existence of KC policies and practice guidelines. KC advances the role of the parent in the care, improved outcomes, and survival of preterm infants. Despite mounting evidence of its effectiveness, scale-up and sustainability remain to be issues in its implementation [48, 49].

This paradigm shift in neonatal caregiving requires a shift in NICU culture. In order to successfully implement KC, caregiving practices, attitudes, and health system priorities need to align to promote its practice. We found that each NICU was on a distinct path through the paradigm shift and that the broad social, cultural, and environmental contexts of a particular NICU affect the uptake of health interventions like KC [16].

To varying degrees, BC’s NICUs are shifting from caregiving driven by HCPs and technology to a model that embraces FCC principles, of not separating parents and infants, increased parent presence, and the idea of KC as a place of care and neonatal therapy and not just a ‘nice to-do’. As each NICU works toward this new model of care, staff must navigate the need for change, the environment and space provided, and HCPs’ attitudes regarding the changing model of care.

The scale-up of KC relies heavily on both parent engagement and HCP support [50]. The uptake of KC aligns with FCC principles in the NICU. Each model of care would benefit from the implementation of the other as both require parent presence in order to be fully realized. Some HCPs spoke about FCC principles while others did not recognize the changing practices of the NICU as FCC.

A key finding from this study is that space and place can affect how KC is practiced. The physical footprint and design of the NICU influences HCPs’ beliefs about the feasibility of KC and whether parents are able to hold their infant in KC. Supporting a shift toward a NICU that has welcoming and comforting spaces is not easily accomplished but it is an important component of the paradigm shift towards FCC and acceptance of KC as a continuous and prolonged versus intermittent practice for short periods of time [13]. Reports of space and design being a barrier to KC and FCC are consistent with other studies [50–52]. Physical space as well as the amenities within the space such as a bed, couch, or comfortable chair support a healing NICU environment. The healing environment encompasses the physical space for families, patients, and staff, as well as the sensory environment that protects the infant’s brain. The environment, combined with a place to rest to be in close proximity to their infant, signals to parents that they are welcome and acknowledges to both staff and parents that parent presence is important [24, 53]. HCPs spoke of the mixed signals families receive from seeing restricted NICU spaces, inappropriate chairs for KC, and lack of places for them to sleep. HCPs were conflicted in their actions and communication to support KC and felt that the environment did not support parental presence or prolonged, continuous KC. A study of two Swedish NICUs found that staff attitudes about KC were more positive in environments that enabled parents to be with their infant at all times [31]. This would be true in our study as well. In our context it would also be important to ensure that the differences between spaces and resources in different NICUs are reduced and the support for families is equitably distributed in order for both staff and parents to not feel that some NICUs are better able to support KC than others.

A recent study that compared parent presence and KC in SFRs to open-bay NICUs found that parent presence in SFRs was three times higher than in the open-bay NICUs and more KC was practiced in SFRs [54]. Prolonged KC is best supported when parents are invited and welcomed into the care setting and understand their role in the NICU [16]. Supporting parents may mean ensuring that those who may face additional barriers such as language have appropriate translated resources.

Our study reiterates the essential role that nurses and other NICU HCPs play in increasing the prevalence of KC [55]. The role of the HCP in the NICU is part of the same paradigm shift in which they find themselves moving from the role of a ‘doer’ toward being a ‘supporter, mentor, and educator’ [56]. NICU nurses were reported as vital to the increased uptake of KC; assisting nurses to realize and understand their changing role will help with this transition.

One approach to overcoming the environmental barriers to KC practice is to support NICUs to find creative solutions to work with what they have. For example, NICUs that are open-bay design cannot immediately change their footprint and design. Instead of complaining that KC is impossible in such conditions, it may be more helpful to ask, ‘how can we scale-up KC given the environmental limitations’? Addressing resource-related barriers can be difficult and may need broader health system changes as well as policy and administrator support [55], but these concerns are not insurmountable.

An essential component of KC is its prolonged and continuous practice. Some HCPs did not support shifting the location of care from the incubator to the parent’s chest and considered prolonged, continuous KC either inappropriate or not feasible. Moving toward this practice change is further limited by conflation of the terms KC and skin-to-skin care and a lack of understanding of the benefits of KC. Skin-to-skin care is often described as intermittent skin-to-skin holding whereas KC is understood as a continuous method with mutually supportive components [31]. The continued misunderstanding of the term skin-to-skin care restricts the ability for NICUs to establish KC as a standard of care. To truly embrace and believe in KC as a standard of care, HCPs must also support the other components of the model (which includes exclusive breast milk and breastfeeding, and appropriate support and follow-up for the infant and family once at home).

Previous studies confirm that staff ‘buy-in’, motivation, and ambivalence are barriers to KC [31, 43]. One study noted that HCP ambivalence towards skin-to-skin care practices comes from “a complex interplay of beliefs, norms, and evidence.” (p.1) [57]. Our study confirms previous research that providing KC is influenced to some degree by HCPs’ beliefs and attitudes rather than simply the scientific evidence. It was clear to us that HCPs’ clinical practices did not always align with what was taught by NICU educators, outlined in existing policy guidelines (when available), and/or best available evidence. Another study also noted the existence of NICU ‘care cultures’ in which the “care beliefs of the bedside neonatal nurse combined with the NICU team members have an impact on the provision of care practices available to the mother and infant” (p. 2) [58]. We found evidence of such care cultures within the NICUs visited. The value of KC was notably expressed through HCPs’ beliefs and embedded routine NICU care practices; these informed the collective NICU culture and shaped the personal philosophy and beliefs of the HCP.

The fear and lack of confidence/skills that many HCPs have in moving babies with lots of tubes/lines appears to be a significant barrier to KC. Previous studies reported that HCPs fear causing accidental extubation or arterial or venous line dislodgement [41, 42, 59]. Individual experience or training with infants in these circumstances increased confidence whereas HCPs who did not receive training in KC with CPAP reported they would not feel comfortable offering KC to such infants. Another study also found that fear and lack of knowledge and training were inhibiting factors. Consistent unit specific messaging, policy and practice guidance, and appropriate training—including simulation—and education on current evidence may improve comfort levels and shift HCPs’ beliefs in the efficacy and appropriateness of KC [60].

Our study also highlights the unique and important role of HCPs in shaping the relationship between infants and their parents [61, 62]. HCPs may act as ‘gatekeepers’ of infant care and KC. HCPs reported that they determine if and when infants are stable enough to engage in KC as well as whether parents are ready for KC. HCPs also reported reluctance to offer 24/7 KC, fearing this may burden parents and cause them to feel guilty if they were unable to provide KC for prolonged periods of time. These decisions were frequently made based on an individual HCP’s preferences as opposed to a discussion with the healthcare team, clinical criteria, or parental decision. Other studies note similar findings that variability and inconsistency in practice continues in part because the practice of KC is left up to a HCP’s discretion and is highly influenced by individual experience, comfort, and clinical judgment [63, 64].

Many HCPs commented that an explicit KC policy would provide a rationale for practice change and assist in standardizing practice. Recommendations from an international KMC workshop and existing evidence suggest that KC become the universal standard of care for all infants in the NICU [65]. Development of a policy may help to address HCPs’ fears of causing instability and their lack of comfort associated with transferring infants into KC that was noted in both level 2 and 3 NICUs in our study. HCPs noted that a policy would provide evidence-based information to support clinical decision-making. A recent study found that a ‘KC pathway’ was effective at operationalizing and translating evidence into practice and providing a unit standard of care. Multidisciplinary champions also helped by empowering staff and facilitating project momentum and supporting creative communication strategies. After recognizing their powerful role in providing safety during KC, in addition to the KC pathway, an education video for NICU nurses was helpful in shifting their acceptance of the research evidence [64].

Embracing the KC model of care also addresses the barriers established by NICU routines and practices. Parents’ access to the NICU continues to be a barrier to KC; some NICUs continue to maintain traditional “visiting hours” for parents. While many NICUs supported parents to be present, in some other units space and design limitations prohibited 24/7 family presence. NICUs with visiting hours were all open-bay design and staff reported shared common challenges maintaining confidentiality, managing shift change and patient handover, and coping in crowded spaces. Positioning parents as ‘visitors’ and restricting access to their infants undermines their parental rights and the rights of the child and parents’ important role in their infant’s care and decision making [66]. In studies of NICU parents’ perspectives on KC, parents reported an emotional toll from not having immediate access to their infant when there were visiting hours or when no accommodation was provided [55].

Parental presence in the NICU is crucial to the implementation of both FCC principles and the KC model of care. According to one author, “when parents are invited - they are both able and willing to provide care and comfort to their infant 24/7 if the physical environment and staff attitudes allow parents’ unrestricted presence and to contribute actively in care of their baby” (p. 1709) [67]. HCPs reported several structural factors that act as barriers and enablers to parental presence such as availability of paid parental leave, transport vouchers, and hot meals. HCPs suggested that the extent to which these barriers impact family presence varies but clearly parents with more financial and familial resources will overcome these barriers more easily than those without. A study of mothers’ experiences of providing KC in the NICU found that structural barriers such as the expenses associated with travelling to the NICU and paying for accommodation cause significant stress [55]. In some countries, like Sweden, the United Nations Convention on the Rights of the Child is now law and establishes the legal obligation and fundamental right of children not to be separated from their parents when hospitalized [68]. This would apply to the NICU and their responsibility to provide a place for a parent to stay overnight with their infant.

We believe that it is possible to implement KC as a model of care in BC’s NICUs if key steps are taken to support a FCC philosophy and work toward NICU cultures that embrace the practice. We recommend the following strategies to address the specific challenges NICUs may face in their local context. These are best achieved when health systems, hospitals, leadership, HCPs, and parents collaborate to shift the neonatal model of care.

Recommendations for the implementation of a KC model of care in BC NICUs:

1. Provincial and local health systems prioritize FCC principles and KC practices
2. Develop and disseminate a provincial practice guideline for KC in the NICU
3. Distribute supportive resources and provide education in multiple languages to families in all NICUs
4. Increase knowledge and awareness of KC through HCP education modules and resources
5. Create welcoming and developmentally supportive environments for infants and parents
6. Provide education and support to HCPs in order to understand the needs of parents and how to engage with parents about the importance of KC
7. Create provincial indicators for monitoring and documenting KC

## Limitations

Several limitations of this study should be noted. While every effort was made to interview HCPs in private rooms, it was not always possible due to staffing levels or time constraints. The interviews sometimes took place in the NICU or at the bedside where other HCPs and parents were present and this may have influenced the participant’s comfort level with respect to expressing their true opinions and experiences openly. While purposive sampling sought to identify participants with rich expert knowledge about KC in their respective NICU, this was achieved through snowball sampling the majority of the time, thereby introducing selection bias and alternative perspectives may have been missed. When long distance travel took place on a specific date and the selected participants were unable to speak to us, any available staff member was asked to be interviewed by the NICU educator or manager, this may have further influenced HCP responses due to the reporting structures Staff who volunteered to be interviewed may have been more willing to share their perspective on KC than others who refused or did not volunteer to be interviewed; the perspectives of those not willing to be interviewed may have differed. In addition one interviewer was previously employed as a neonatal nurse at one of the participating hospitals; she may have been known to staff in this NICU. This may have influenced some participants’ responses, however it was felt that there was value in having a neonatal nurse with recent experience in order to understand the clinical care and terminology.

## Conclusions

KC is a complex intervention, with unique barriers and enablers. In BC, there is no ‘one size fits all’ approach to address barriers that limit or promote KC practice, given practice is situated in the context of each individual NICU. We found some universal barriers and enablers but observed that these can vary based on the factors within the NICU (i.e., physical space, staff attitudes, and allocated resources). Understanding the successes and challenges experienced by the various NICUs will help shape the development of practical and responsive recommendations for changes required at various levels of the healthcare system.

This study highlighted the importance of the role of the HCP in managing change and advancing evidence-based practice. For this model of care to be actualized, HCPs require understanding and awareness of how current care and practice impairs preterm infant outcomes. HCPs can be leaders for change within their institution, advocates for infants, educators and supporters for parents, and a resource and mentor to other staff. Harnessing the power of champions in each NICU may also help support further implementation and make KC a priority in the health system. HCP champions may be effective in garnering support from hospital administrators to place more resources towards ensuring parent presence and close parent-infant relationships.

Ultimately, province-wide implementation of FCC principles as the philosophical foundations for care in the NICU will advance KC as the standard of care across BC. Creating a family-centered environment should be the goal for every NICU in the long-term but making small changes in the short-term may lead to greater satisfaction and more successful implementation. By extension, the mother-baby dyad must be protected and hospitals need to practice zero separation starting from birth to the maternity units and the NICU. A provincial mandate is required to ensure that parents are not separated from their infants after birth in order to reinforce that an infant has the same right as a child to be with their parent during hospitalization. To continue to strengthen KC, HCPs, policy makers, and hospital leaders will need to assess their current practices, routines, and cultures. Within the local context, each NICU will require a shifting of their policies and roles that may be improved by changes in the physical space and design towards one that supports parent presence, empowerment, and engagement in their infant’s care.

We are early in our journey to maximizing the practice of KC and for infants and families to fully realize its therapeutic benefits. We no longer need to ask the question “why should we do KC?” but move beyond this and challenge ourselves to make KC the standard of care across the province and understand what do we need to do to achieve this goal and ask a new question, “What is holding us back?”

## Supplementary information

**Additional file 1.** Healthcare provider interview questions.

**Additional file 2.** Neonatal Daily classification table to inform readers about the levels of NICU care in BC.

## Data Availability

The data that support the findings of this study are available on reasonable request from the corresponding author. The data are not publicly available due to them containing information that could compromise research participant privacy and consent.

## Abbreviations

BC: British Columbia
HCP: Healthcare Provider
KC: Kangaroo Care
NICU: Neonatal Intensive Care Unit
SFR: Single Family Room
FCC: Family Centered Care
