# Supplementary material for: What is stopping us? An implementation science study of kangaroo care in British Columbia’s neonatal intensive care units
Source: BMC Pregnancy Childbirth. 2021 Jan 12;21:52. doi: 10.1186/s12884-020-03488-5 (PMC7805090; doi:10.1186/s12884-020-03488-5)
Supplement: Supplementary file 1 — Additional file 1. Healthcare provider interview questions. [file 12884_2020_3488_MOESM1_ESM.docx]

**Date:**

**Key stakeholder:**

**Research team member:**

**Hospital:**

**HISTORY**

1. Please tell me your role and how long you have worked in that role? When did you come to work at __________________?
2. Does your NICU currently practice Kangaroo Care in your unit? *(If the unit, uses the term skin-to-skin care – explore what this means, how it might be different of the same. Was there a reason skin-to-skin was chosen?)*
3. What is the length of time that Kangaroo Care is recommended to be practiced?
4. What do you think the average amount of time most families practice Kangaroo Care?
5. What is the history of Kangaroo Care in your hospital? *(Was there a specific occasion or meeting where the decision to implement or strengthen KC/skin-to-skin was taken? Who would be involved in this type of decision making? Did you receive funding from the health authority or hospital to make this a priority?)*
   1. Has there been previous efforts to introduce Kangaroo Care into the unit?
   2. How does staff support Kangaroo Care currently?
   3. What is going well?
6. What do you understand (how would you describe the benefits or importance of KC) about the benefits of skin-to-skin contact and Kangaroo Care?
7. In your unit or hospital – who (what professions/groups of people) would be involved in supporting and strengthening Kangaroo Care?
   1. What are the training and/or development and support needs of those whose role it is to either initiate or support Kangaroo Care?

FAMILIES

1. How is Kangaroo Care introduced to parents in the NICU?
   1. When is the first time they hear about it?
   2. How do you introduce Kangaroo Care to parents? When would this occur?
   3. Are any parents excluded from practicing Kangaroo Care?
   4. Have you ever experienced any religious or cultural practises that would negatively or positively influence a parent to practice Kangaroo Care? How was it handled?
   5. On a scale of 1-5, where 5 is very confident and 1 is not very confident, how confident do you feel about your ability to teach and /or support Kangaroo Care with NICU parents?

**Kangaroo Care DEFINITION and BARRIERS/ENABLERS**

The Kangaroo Care program will be using the following definition for Kangaroo Care which has 3 important components:

1. Skin-to-skin contact between baby and parents (early, continuous and prolonged – ideally 24 hours a day)
2. Exclusive breastfeeding (or access for preterm infants, access to breast milk and support with the initiation and maintenance of breast milk)
3. Support for the mother, baby, and family without separation which may include support for earlier discharge home once mother and family are capable to taking over the care of their baby.
4. What are your thoughts on this definition? Do you see any issues that might arise with introducing this definition to staff members? To parents?

In order to best support and strengthen Kangaroo Care we are trying to understand the current barriers and enablers of Kangaroo Care. We will explore these during the next few questions.

1. Can you identify any possible barriers to increasing the length of time that parents spend doing Kangaroo Care?
2. Can you identify any concerns that may arise with the 3^rd^ component? Can you identify any barriers to the possibility of earlier discharge?
3. What are the current discharge criteria for infants discharged from the NICU?
4. Are there any other barriers (physical space, environment, culture) can you think of that might prevent a successful scaling up of Kangaroo Care at this hospital?
5. Literature cites that one barrier for NICU staff are the different definitions for infant eligibility? Would this be true in your hospital? What are the eligibility criteria for your hospital? Do you think this can be changed?
6. Do you have any concerns regarding the practice of Kangaroo Care? Have there been any issues in terms of safety of the infant and/or parent?

**TRAINING/EDUCATION/INFORMATION SHARING**

1. What types of education or training (awareness) does staff currently receive about Kangaroo Care or skin-to-skin contact? (nursing, medical)
2. Do you think nurses currently support the practice of Kangaroo Care in the NICU? How do they or don’t they?
3. Do you think physicians currently support the practice of Kangaroo Care in the NICU? Can you please provide examples?
4. Are there any other disciplines that might play a key role in supporting KC (lactation consultants, OT, PT, RT)? How might they be supportive or not? How could we provide support to them to engage others?

**CLINICAL**

1. Do you currently use a wrap when you are supporting parents to practice?
   1. What kind of wrap?
   2. What has worked for you?
   3. Do you think parents like the wrap?
2. When mothers are supported to breastfeed is Kangaroo Care mentioned? Are they linked?
3. The goal of Kangaroo Care is to have the parent hold their baby 24 hours a day. In order to do this, it is expected that the parent will need to rest.
   1. Does your unit have safe sleep guidelines? How is it given to parents?
   2. What do you recommend about co-sleeping?
   3. Do you see this as a potential barrier for staff to support Kangaroo Care?
4. Admission: When moderate to late preterm infants are admitted to the nursery, are they ever transferred in skin to skin with the mother or partner? Who decides if this is possible?
5. Are parents’ encouraged to practice Kangaroo Care or skin-to-skin contact after discharge?
6. On a scale of 1-5, where 5 is very important and 1 is not important, how important do you see Kangaroo Care in terms of overall NICU care? (for example, best outcomes, improved parent and infant health, earlier discharge, bonding etc)

**SUMMARY**

1. Is there anything else about Kangaroo Care that you would like to tell me about or comment on? Any other barriers or enablers of Kangaroo Care in your hospital that you can think of?
2. Would you be interested in engaging in a 15 minute follow-up interview in about four months?
